# Comparative Analysis of the Cultured and Total Bacterial Community in the Wheat Rhizosphere Microbiome Using Culture-Dependent and Culture-Independent Approaches

**DOI:** 10.1128/Spectrum.00678-21

**Published:** 2021-10-20

**Authors:** Sameh H. Youseif, Fayrouz H. Abd El-Megeed, Ethan A. Humm, Maskit Maymon, Akram H. Mohamed, Saleh A. Saleh, Ann M. Hirsch

**Affiliations:** a Department of Microbial Genetic Resources, National Gene Bank (NGB), Agricultural Research Center (ARC), Giza, Egypt; b Department of Molecular, Cell & Developmental Biology, University of California–Los Angeles (UCLA), Los Angeles, California, USA; c Agricultural Microbiology Research Department, Soils, Water and Environment Research Institute, Agricultural Research Center (ARC), Giza, Egypt; d Molecular Biology Institute, University of California–Los Angeles (UCLA), Los Angeles, California, USA; University of Massachusetts Amherst

**Keywords:** rhizosphere, microbiome, culturability, OTU, wheat, bacterial community, arid soils, microbial communities, rhizosphere-inhabiting microbes

## Abstract

Rhizosphere and root-associated bacteria are key components of crop production and sustainable agriculture. However, utilization of these beneficial bacteria is often limited by conventional culture techniques because a majority of soil microorganisms cannot be cultured using standard laboratory media. Therefore, the purpose of this study was to improve culturability and investigate the diversity of the bacterial communities from the wheat rhizosphere microbiome collected from three locations in Egypt with contrasting soil characteristics by using metagenomic analysis and improved culture-based methods. The improved strategies of the culture-dependent approach included replacing the agar in the medium with gellan gums and modifying its preparation by autoclaving the phosphate and gelling agents separately. Compared to the total operational taxonomic units (OTUs) observed from the metagenomic data sets derived from the three analyzed soils, 1.86 to 2.52% of the bacteria were recovered using the modified cultivation strategies, whereas less than 1% were obtained employing the standard cultivation protocols. Twenty-one percent of the cultivable isolates exhibited multiple plant growth-promoting (PGP) properties, including P solubilization activity and siderophore production. From the metagenomic analysis, the most abundant phyla were *Proteobacteria*, *Actinobacteria*, *Chloroflexi*, *Bacteroidetes*, and *Firmicutes*. Moreover, the relative abundance of the specific bacterial taxa was correlated with the soil characteristics, demonstrating the effect of the soil in modulating the plant rhizosphere microbiome.

**IMPORTANCE** Bacteria colonizing the rhizosphere, a narrow zone of soil surrounding the root system, are known to have beneficial effects in improving the growth and stress tolerance of plants. However, most bacteria in natural environments, especially those in rhizosphere soils, are recalcitrant to cultivation using traditional techniques, and thus their roles in soil health and plant growth remain unexplored. Hence, investigating new culture media and culture conditions to bring “not-yet-cultured” species into cultivation and to identify new functions is still an important task for all microbiologists. To this end, we describe improved cultivation protocols that increase the number and diversity of cultured bacteria from the rhizosphere of wheat plants. Using such approaches will lead to new insights into culturing more beneficial bacteria that live in the plant rhizosphere, in so doing creating greater opportunities not only for field application but also for promoting sustainability.

Wheat (Triticum aestivum L.) is one of the most important food crops in the world. It is one of the key staple crops for global food security, providing 20% of the daily requirement of calories and protein in human nutrition (1). Current agricultural practices focus on improving wheat yield by changing the plant microbiome to improve its nutrition and resistance to pathogens and abiotic stressors (2, 3). However, plants also recruit and “engineer” their rhizobiome toward beneficial root symbionts (2) via the synthesis and secretion of specific root exudates (4). For example, the wheat rhizosphere consists of numerous beneficial plant growth-promoting rhizobacteria (PGPR) that contribute directly or indirectly to the growth and fitness of plants, by providing phytohormones, solubilizing nutrients, fixing nitrogen (N_2_), employing biocontrol mechanisms against phytopathogens, and alleviating abiotic stresses (5–7). *Bacillus*, Pseudomonas, and *Stenotrophomonas* species are some examples of well-recognized PGPR that predominate in the wheat rhizosphere (5, 7, 8). Hence, it is very important to develop new methods and media for exploring cultivable PGPR, a possible key to future microbiome engineering solutions in sustainable agriculture (9).

Currently, only a minor portion of viable cells of microbial populations in nature can be cultured under laboratory conditions (10); indeed, the vast majority of microorganisms remain uncultured (11). This phenomenon is known as the “great plate count anomaly” (12). The recent development of sequencing technologies, including high-throughput sequencing based on 16S rRNA gene amplicons, has enabled researchers not only to provide new insights into uncultured microorganisms, but also to explore their potential functions in the environment (13, 14). Moreover, environmental microbes are important for sustainable agriculture, and culturing the “unculturable” is critical for their application to crops. Accordingly, many attempts have been made to develop more efficient methods of isolating as well as growing recalcitrant soil bacteria (15–17). These strategies include cultivation platforms that mimic natural environments (15), changing the type of solidifying agents (18), and modifying both growth media (17) and culture conditions (19).

Gelling agents are frequently used to solidify liquid culture media, usually in cultivating microbes of a single species. Historically, agar has been the predominant gelling agent in microbiological research, but a shortcoming in the preparation of agar-solidified medium is the difficulty in culturing microorganisms from diverse habitats because of how the agar is prepared (17, 20). Autoclaving agar together with phosphate generates hydrogen peroxide (H_2_O_2_), which limits both growth and colony formation of many microorganisms (20), whereas the separate sterilization of agar and phosphate improves the culturability of a wide range of microorganisms, including slow-growing bacteria (17), anaerobic bacteria (21), and rare actinobacteria (22), due to reduced H_2_O_2_ production. In addition to the inhibitory effect of the peroxides, agarase produced by some bacteria can interfere with agar solidification and reduce the number of discrete colonies formed on a plate (23). For these and other reasons, interest in exploring alternative gelling agents to improve microbe culturability has grown (24). Gellan gums such as Gelrite and Phytagel are water-soluble, nitrogen-free polysaccharides produced by *Sphingomonas* spp. (25). They do not contain phenolic compounds, which are toxic to several bacterial species (26). Gellan gum has become a significant substitute to replace agar for cultivating recalcitrant microorganisms (18, 27), which enables their isolation (26, 27). Although gellan gum produces some H_2_O_2_ when autoclaved together with phosphate buffer, its concentration is significantly lower than the concentrations produced in agar-based medium preparations (28).

Based on previous reports, we hypothesized that testing new medium preparations, including alternative gelling agents, would increase the culturability of soil microbiome organisms. We studied how the gellan gums Gelrite and Phytagel compared with agar affected the cultivability and diversity of bacterial communities, especially the PGPR, in the wheat rhizosphere microbiome isolated from three different soils from Egypt. We also evaluated the impact of the separate sterilization of phosphate and solidifying agents on the isolation and colony formation of cultivable bacteria. In parallel, we assessed the bacterial diversity in the wheat rhizosphere using cultivation-independent metagenomic analysis to understand better the influence of soil on microbial diversity.

## RESULTS

### Physicochemical properties of soil samples.

The three soil samples displayed differences not only in their mineral and physical compositions but also in pH, salinity, and organic matter (see Table S1 in the supplemental material). According to FAO-accepted classification (29, 30), the soil from the Luxor site is a clay loam, slightly alkaline (pH 8.00), and contains 52.20 ppm of total soluble N and 0.72% organic matter. The soil from the Minya site has a sandy structure, is slightly alkaline (pH 7.93), and has 14.30 ppm of total soluble N and 0.16% organic matter. The soil from the Nubaria site is a sandy loam and also calcareous (18.80% CaCO_3_). In addition, it is moderately alkaline (pH: 8.51) and contains 16.40 ppm of total soluble N and 0.21% organic matter. Although the three soil samples are all classified as nonsaline, the soil sample from the Nubaria site is more likely to be affected by salt stress (electrical conductivity [EC], 0.84 dS m^−1^) than the soil samples collected from the Luxor and Minya sites (EC, 0.62 and 0.33 dS m^−1^, respectively).

### Comparison of CFU counts on different medium preparations.

**FIG 1:**
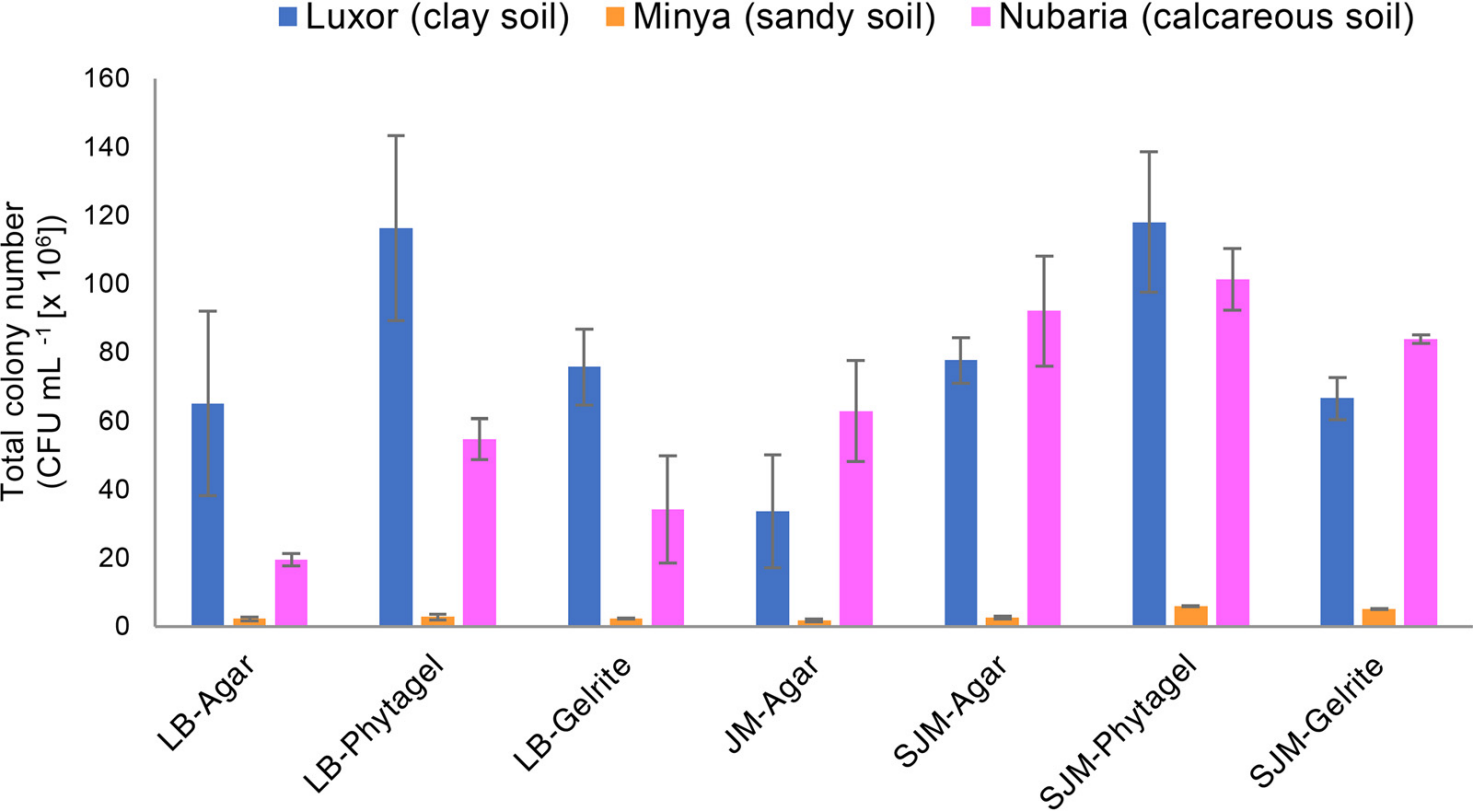
Effect of isolation media and gelling agents on the total colony number of bacteria recovered from the wheat rhizosphere microbiome.

Different strategies were applied to increase the magnitude of isolated bacteria from the three samples of wheat rhizospheric soil. Bacterial abundance was determined by direct cell counts on 7 medium preparations made with three solidifying agents (agar, Gelrite, and Phytagel) and based on two different growth media: LB medium and Jensen medium (JM) (Fig. 1). Although the numbers of CFU varied according to the soil type and growth medium, the highest numbers of CFU were obtained on substrates solidified with Phytagel under all tested conditions. For LB medium, the highest numbers of CFU were recorded using Phytagel followed by Gelrite and agar, respectively. The highest numbers of CFU ([116.3 ± 27.0] × 10^6^ cells ml^−1^) were acquired on LB Phytagel medium for clay soil collected from the Luxor site, whereas the lowest numbers of CFU ([2.08 ± 0.51] × 10^6^ cells ml^−1^) were found on LB agar medium in the sandy soil collected from the Minya location. For Jensen medium, a lower cultivability in all tested soils was obtained when phosphate and agar were autoclaved together (JM agar), indicating the inhibitory effect of the generated H_2_O_2_ on bacterial growth.

### Diversity of cultivable bacteria associated with wheat rhizosphere microbiome using different medium preparations and the effects of soil type.

A total of 177 different pure colonies were selected according to their morphology (see Table S2 in the supplemental material), including 41 pure colonies from clay soil (Luxor), 84 pure colonies from calcareous soil (Nubaria), and 52 pure colonies from sandy soil (Minya). However, 11 bacterial colonies failed to be subcultured on their respective medium or any other media. The remaining 166 isolates (85 from LB medium and 81 from Jensen medium) were cultured on their respective media for DNA isolation and subsequently sequenced using the 16S rRNA gene to determine their phylogenetic positions.

**FIG 2:**
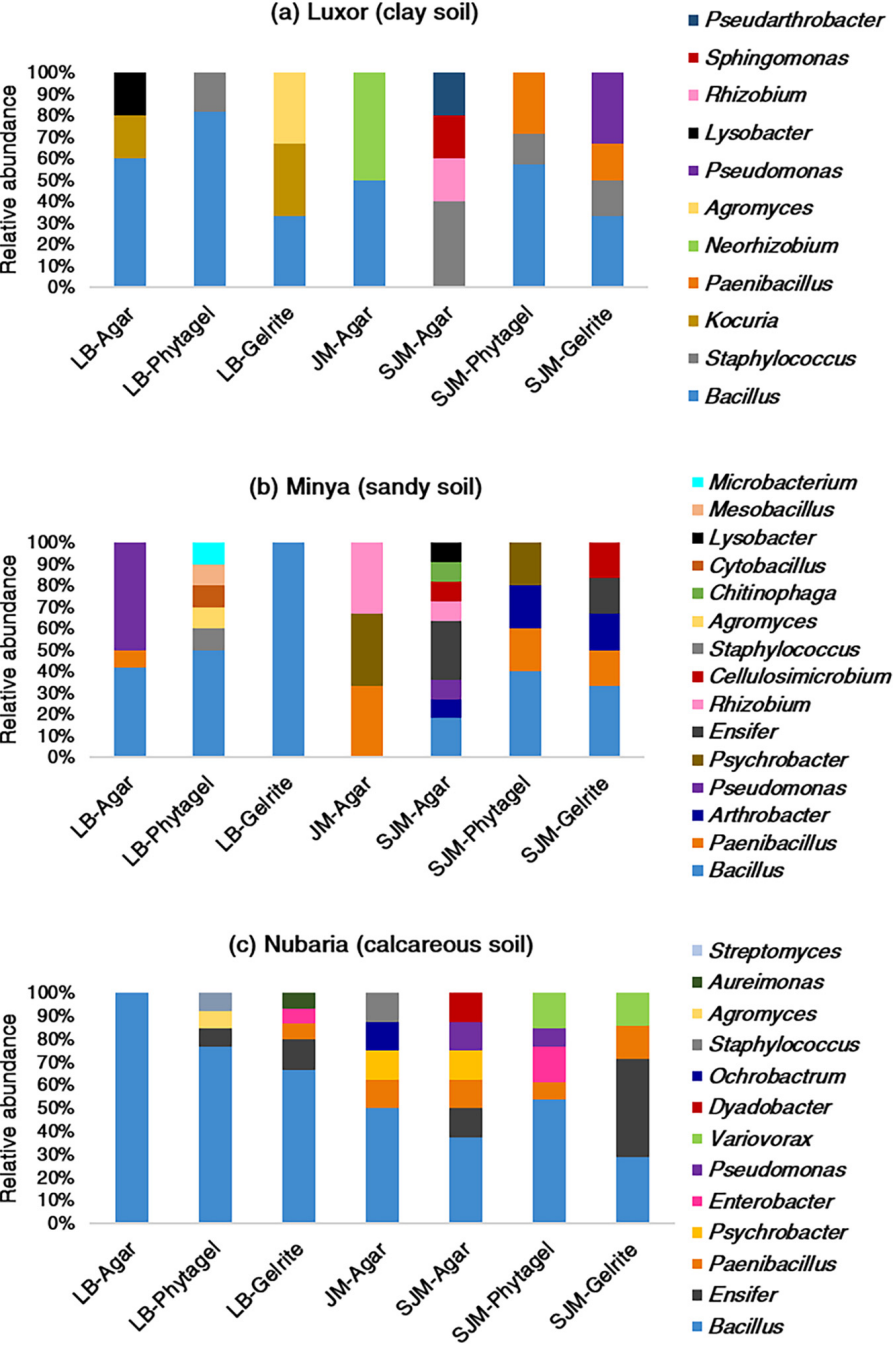
Bacterial taxonomic composition of cultivable fractions in the wheat rhizosphere microbiome collected from (a) Luxor, (b) Minya, and (c) Nubaria sites.

Generally, the structure of the cultured fractions of the bacterial communities was affected by a number of factors, including isolation medium, cultivation protocol, gelling agents, and soil type (Fig. 2 and Table 1). For LB medium, *Bacillus* was the predominant genus cultured in each of the three solidifying agents. We found that LB substrates solidified with Phytagel and Gelrite resulted in a higher diversity of bacterial genera in sandy and calcareous soils, respectively. However, growth on agar and Gelrite resulted in the isolation of more diverse bacterial genera from clay soil compared to Phytagel. In general, most of the actinobacterial genera (for example *Agromyces*, *Microbacterium*, and *Streptomyces*) grew only on LB medium solidified with Phytagel and/or Gelrite. *Bacillus*-related genera also appeared to have a consistently higher relative abundance and diversity on LB medium solidified with Phytagel. Although the gelling gum agents supported the growth of *Aureimonas* and *Ensifer* genera belonging to the *Alphaproteobacteria*, growth of *Lysobacter* and Pseudomonas (*Gammaproteobacteria*) was observed only on LB plates solidified with agar.

**TABLE 1:** Classification of cultivable isolates from agar- and gellan gum-based media

Phylum	Class	Assigned genera	Total isolates (*n* = 166)	No. of isolates cultivable on^*a*^:						
				LB agar (*n* = 32)	LB Phytagel (*n* = 34)	LB Gelrite (*n* = 19)	JM agar (*n* = 13)	SJM agar (*n* = 24)	SJM Phytagel (*n* = 25)	SJM Gelrite (*n* = 19)
*Actinobacteria*	*Actinobacteria*	*Agromyces*	3	0	2	1	0	0	0	0
		*Arthrobacter*	3	0	0	0	0	1	1	1
		*Cellulosimicrobium*	2	0	0	0	0	1	0	1
		*Kocuria*	2	1	0	1	0	0	0	0
		*Microbacterium*	1	0	1	0	0	0	0	0
		*Pseudarthrobacter*	1	0	0	0	0	1	0	0
	*Actinomycetes*	*Streptomyces*	1	0	1	0	0	0	0	0
*Bacteroidetes*	*Sphingobacteria*	*Dyadobacter*	1	0	0	0	0	1	0	0
		*Chitinophaga*	1	0	0	0	0	1	0	0
*Firmicutes*	*Bacilli*	*Bacillus*	88	23	24	12	5	5	13	6
		*Cytobacillus*	1	0	1	0	0	0	0	0
		*Mesobacillus*	1	0	1	0	0	0	0	0
		*Paenibacillus*	12	1	0	1	2	1	4	3
		Staphylococcus	8	0	3	0	1	2	1	1
*Proteobacteria*	*Alphaproteobacteria*	*Aureimonas*	1	0	0	1	0	0	0	0
		*Ensifer*	11	0	1	2	0	4	0	4
		*Ochrobactrum*	1	0	0	0	1	0	0	0
		*Neorhizobium*	1	0	0	0	1	0	0	0
		*Pararhizobium*	2	0	0	0	1	1	0	0
		*Rhizobium*	1	0	0	0	0	1	0	0
		*Sphingomonas*	1	0	0	0	0	1	0	0
	*Betaproteobacteria*	*Variovorax*	3	0	0	0	0	0	2	1
	*Gammaproteobacteria*	Enterobacter	3	0	0	1	0	0	2	0
		*Lysobacter*	2	1	0	0	0	1	0	0
		Pseudomonas	11	6	0	0	0	2	1	2
		*Psychrobacter*	4	0	0	0	2	1	1	0

a JM, Jensen medium prepared by autoclaving phosphates and solidifying agent together; SJM, Jensen medium prepared by autoclaving phosphates and solidifying agent separately.

For the Jensen medium experiments, autoclaving phosphate and agar separately (SJM agar) greatly improved the cultivability of diverse bacterial genera compared to the number of colonies obtained when phosphate and agar were autoclaved together (JM agar). For example, SJM agar medium supported the growth of bacterial genera from the phyla *Actinobacteria* (*Arthrobacter*, *Cellulosimicrobium*, and *Pseudarthrobacter*) and *Bacteroidetes* (*Dyadobacter* and *Chitinophaga*), whereas these genera failed to grow on JM agar medium. Also, we found that the SJM agar medium favored the growth of many proteobacterial genera compared to the JM agar medium. Of note, *Ochrobactrum* (Nubaria) and *Neorhizobium* (Luxor), two alphaproteobacterial genera, were exclusively recovered from JM agar plates.

Comparisons at the genus level of the cultured bacteria obtained using the three different solidifying agents demonstrated that SJM agar substrate resulted in the growth of more diverse genera than did the substrates solidified by Phytagel and Gelrite. For example, members of the phylum *Bacteroidetes* (*Dyadobacter* and *Chitinophaga*) grew only on SJM agar plates. Conversely, *Variovorax*, the only representative of class *Betaproteobacteria*, grew only on Jensen medium solidified with Phytagel and Gelrite.

**FIG 3:**
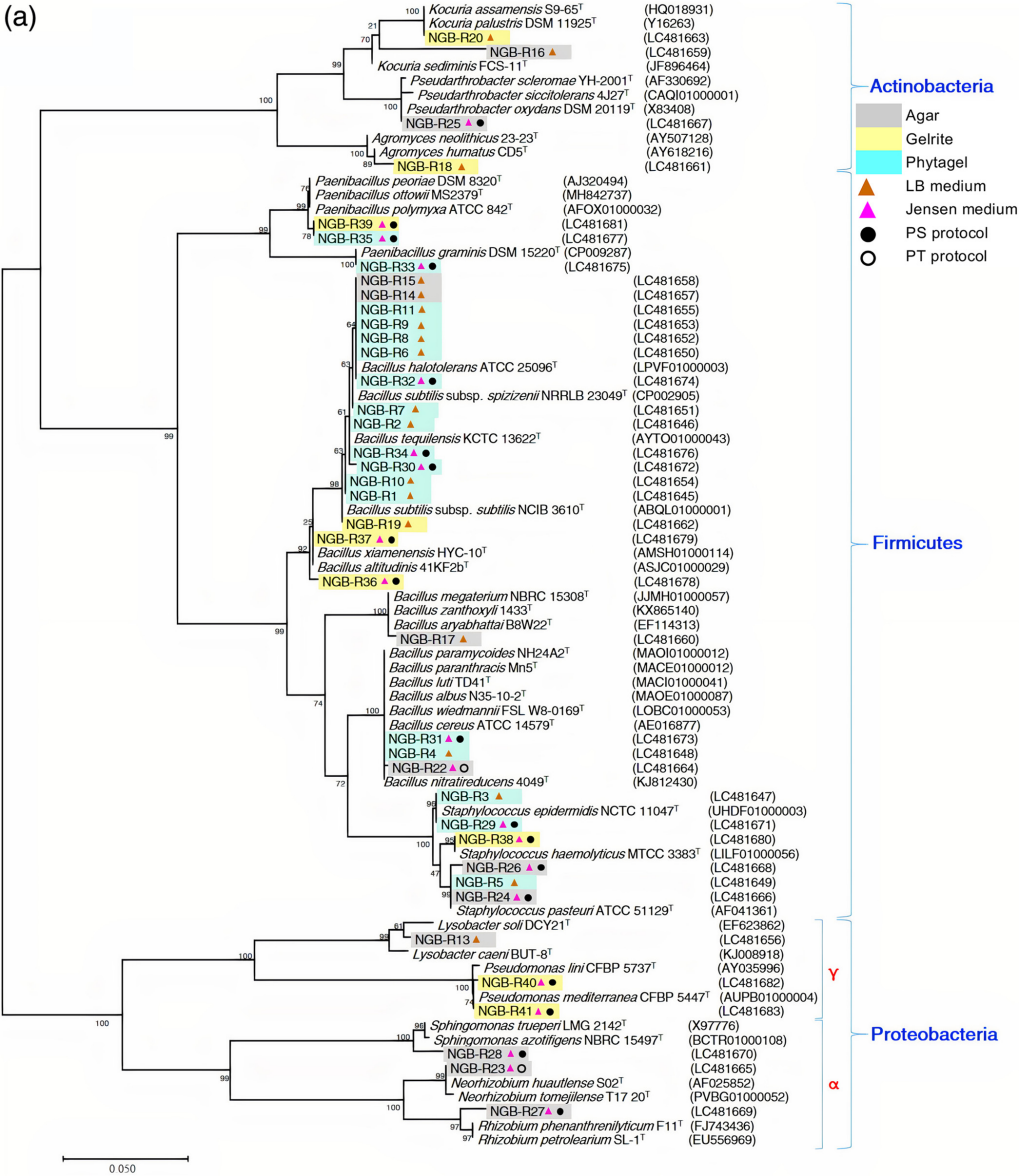

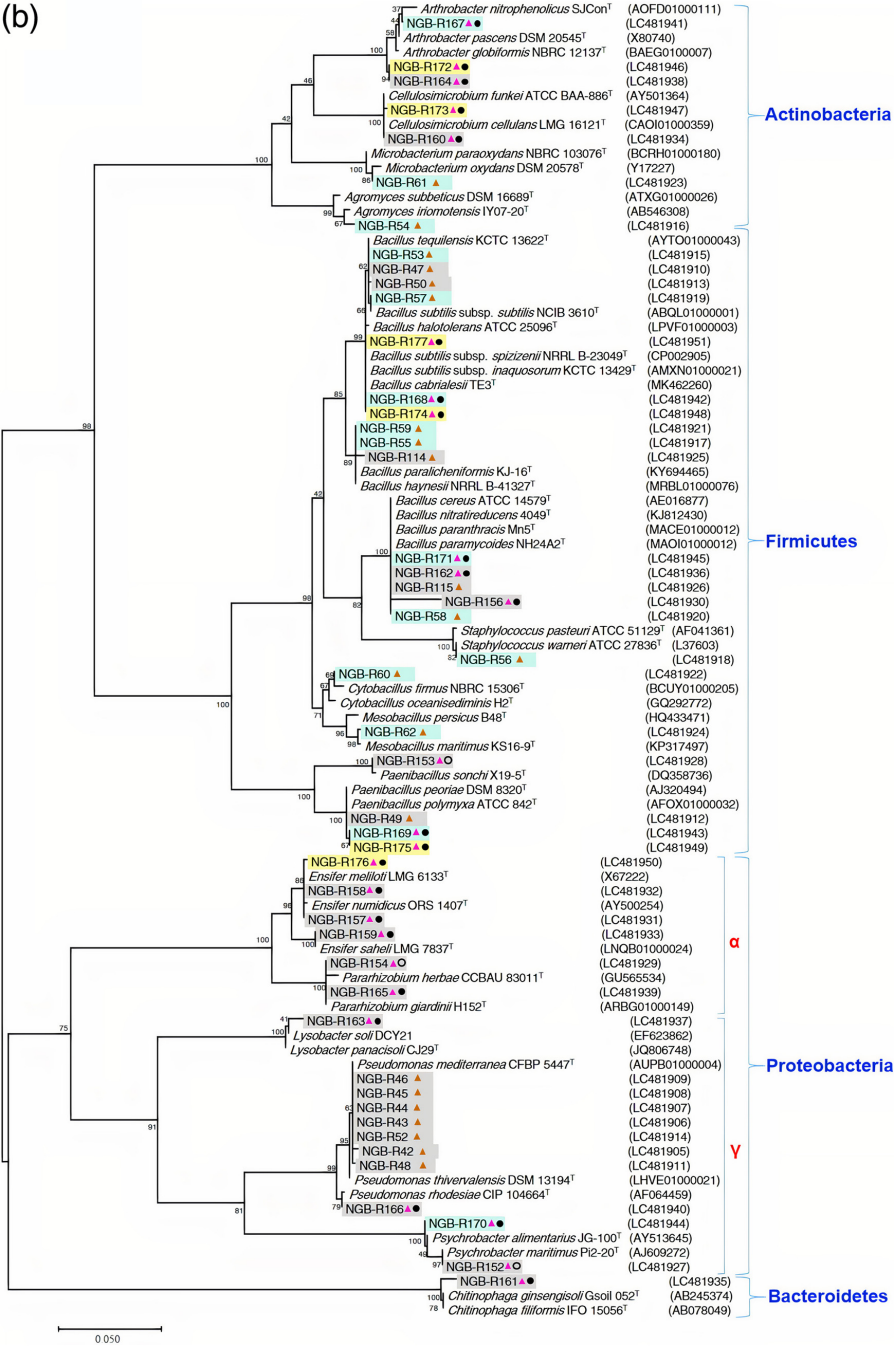

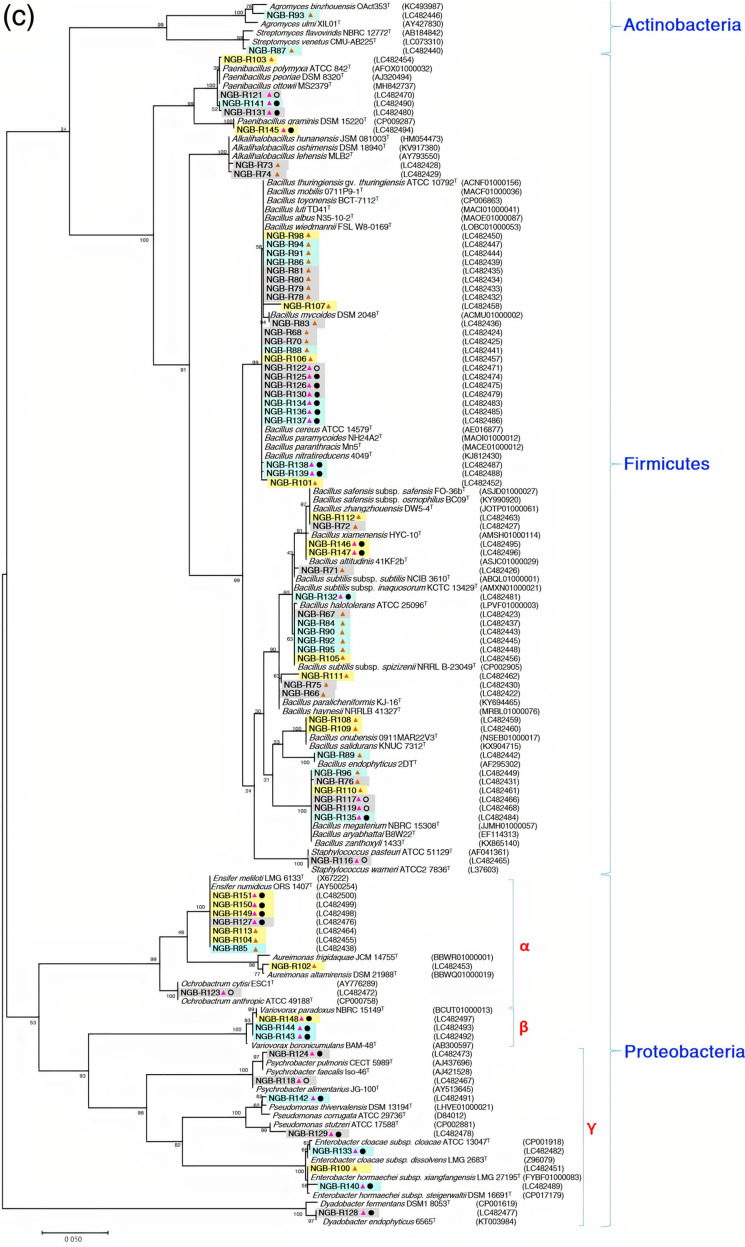
Maximum-likelihood phylogenetic trees based on 16S rRNA gene sequences of cultivable bacterial isolates obtained from (a) clay soil at Luxor site, (b) sandy soil at Minya site, and (c) calcareous soil at Nubaria site. Bootstrap values are indicated for each node (1000 replicates). NGB-R: National Gene Bank-Rhizobacteria. PT protocol (phosphates and gelling agent autoclaved together) and PS protocol (phosphates and gelling agent autoclaved separately).

The phylogenetic analyses of the bacterial communities recovered from the three soil samples (Fig. 3) suggested that cultivation protocol and isolation medium might be stronger drivers of community structure than the type of solidifying agent. For example, despite the soil type, the actinobacterial genus *Arthrobacter* and the proteobacterial genus Pseudomonas grew on JM solidified by the three different gelling agents, but only when the phosphates and gelling agents were autoclaved separately (the PS protocol, where “P” is phosphate and “S” represents “separately”), and not when phosphates and gelling agents were autoclaved together (the PT protocol, where “T” represents “together”). The exceptions were two *Pararhizobium* strains, NGB-R154 and -R165, which grew only on JM solidified with agar and not with other gelling agents, even when the PS or PT protocols were used. The results substantiated the impact of substrate and potentially soil type on the culturable bacterial fractions. For example, isolates of the actinobacterial genus *Agromyces* from clay soil were recovered on LB medium solidified with Gelrite, whereas they grew only on LB substrate solidified with Phytagel if isolated from sandy and calcareous soils.

### Bacterial community analysis in the wheat rhizosphere using the culture-independent approach.

**FIG 4:**
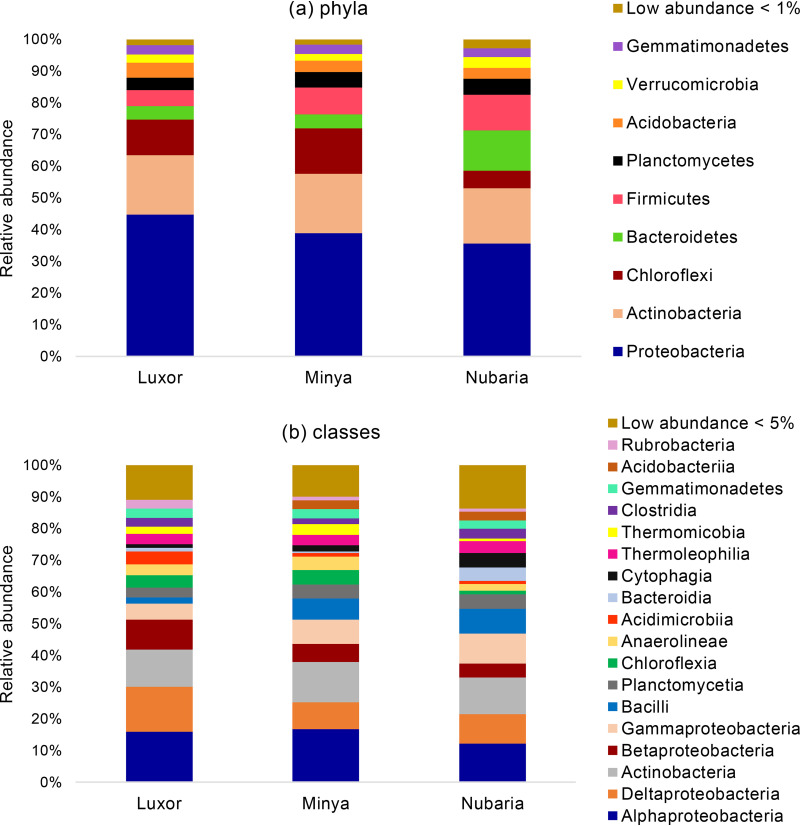
Main phyla (a) and classes (b) of bacteria identified in the wheat rhizosphere microbiome based on the metagenomic analysis; Luxor (clay soil), Minya (sandy soil), and Nubaria (calcareous soil).

Based on the metagenomic analyses, the predominant sequences were represented by bacteria (>90%), and the relative abundances of bacteria in each soil sample were as follows: Luxor, 91.2%; Minya, 93.9%; and Nubaria, 91.5%. A very low percentage of eukaryotic microorganisms was detected in the Luxor and Minya soil samples (<1%), whereas the relative abundance of eukaryotes was higher in the Nubaria sample (6.7%). Compared to the eukaryotes, a low percentage of archaea was detected in the Nubaria soil sample (1.8%), whereas in soil samples collected from Luxor (8.1%) and Minya (5.4%), the percentages were higher. Phylotype classification of the operational taxonomic units (OTUs) indicated that of the 30 bacterial phyla, 21 represented less than 1% of the total OTUs detected in all the soil samples (see Table S3 in the supplemental material). Overall, it appears that the bacterial communities from the three soil samples are compositionally similar (Fig. 4a), with over 35% of the phyla being composed of the phylum *Proteobacteria*: Luxor, 40.8%; Minya, 36.6%; and Nubaria, 32.5%.

The second-most-abundant phylum consisted of *Actinobacteria*, which comprised <15% in each sample: Luxor, 17.2%; Minya, 17.5%; and Nubaria, 15.9%. *Chloroflexi* (4.9 to 13.5%), *Bacteroidetes* (3.9 to 11.7%), and *Firmicutes* (4.6 to 10.3%) were subdominant, but their abundance varied depending on the soil type. For example, *Chloroflexi* were more common in soils from Luxor and Minya (10.2 and 13.5%, respectively) than from Nubaria (4.9%). In contrast, representatives of *Bacteroidetes* and *Firmicutes* more frequently inhabited soils collected from Nubaria (11.7 and 10.3%, respectively) versus soils collected from Luxor (3.9 and 4.6%, respectively) and Minya (4.1 and 7.9%, respectively). In addition, a lower percentage of *Planctomycetes*, *Acidobacteria*, *Verrucomicrobia*, *Gemmatimonadetes*, and *Cyanobacteria* was detected in the three soil samples. At the class level (Fig. 4b; see Table S4 in the supplemental material), the structure of the bacterial community in the three soil samples was dominated by *Alphaproteobacteria* (11.1 to 15.7%), followed by Deltaproteobacteria (7.9 to 13.0%) and *Actinobacteria* (10.4 to 11.9%). Of the subdominants, the relative abundance of the *Betaproteobacteria* class was higher in the Luxor sample (8.6%) compared to the Minya (5.4%) and Nubaria (4.0%) soil samples. Finally, the rhizosphere of wheat plants cultivated in Minya and Nubaria exhibited high levels of *Gammaproteobacteria* (7.2 and 8.7%, respectively) and *Bacilli* (6.2 and 7.0%, respectively), whereas a lower level was detected in the rhizosphere of wheat plants cultivated in Luxor: 4.5 and 1.9%, respectively. At the genus level, we found that the soils from Minya and Nubaria sites had more diverse genera (517 and 592 genera, respectively) than those identified in the Luxor soil sample (464 genera). Because the percentages of most of these genera were very low (below 1% of the detected sequences), the top genera in three soil samples with their percentages are shown in Table S5 in the supplemental material. For example, in the Luxor sample, the most dominant genera were *Pelobacter* (6.4%), “*Candidatus* Nitrososphaera” (5.8%), and *Rhodopseudomonas* (3.2%), whereas *Bacillus* (4.5%), *Arthrobacter* (4.4%), and *Pelobacter* (3.6%) were more frequently detected in the Minya soil sample. The most abundant genera in the Nubaria sample were *Bacillus* (5.3%), *Cytophaga* (3.3%), *Steroidobacter* (3.2%), and *Ohtaekwangia* (3.1%).

### Comparison between culture-dependent and culture-independent analyses.

**FIG 5:**
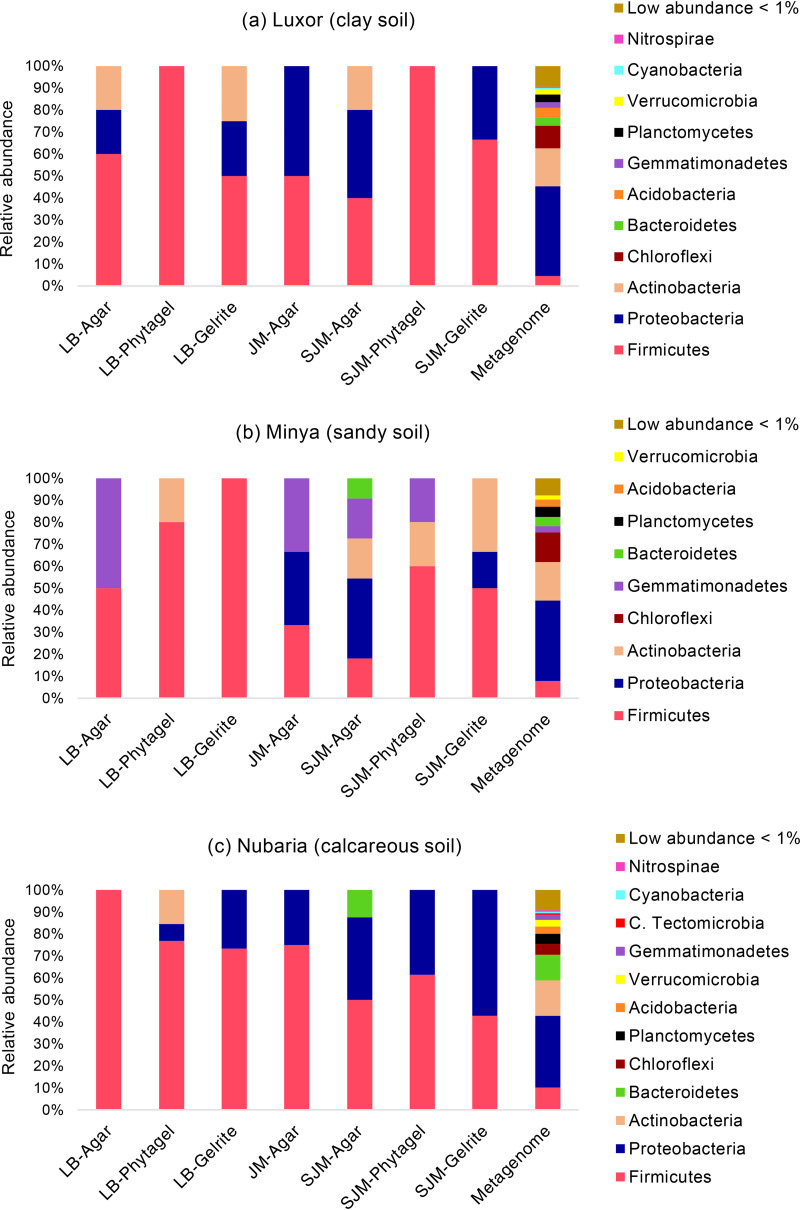
Comparison of metagenomic (culture-independent) and culture-dependent bacteria datasets at the phylum level distributed in wheat rhizosphere microbiome (a) Luxor, (b) Minya, and (c) Nubaria.

To clarify whether modifications of the culture method allowed us to isolate more diverse bacteria, we compared the taxonomic distributions of cultivable fractions from each culture condition preparation with those obtained by metagenomic analyses at the phylum level (Fig. 5) and class level (see Fig. S1 in the supplemental material). According to the results obtained from the three soil types, there were substantial differences in the phylogenetic compositions in the different culture preparations versus those obtained from the metagenomic data set. Of the 70 classes and 30 phyla identified in the metagenomic data sets (Tables S3 and S4), only 7 classes representing 5 phyla were recovered in the culture collection. At the phylum level, there were overwhelming numbers of cultivable *Firmicutes* from the three soil samples (40 to 100%, 18 to 100%, and 43 to 100% in the clay, sandy, and calcareous soils, respectively) compared to the abundances indicated by the metagenomic analyses (4.6, 7.9, and 10.3% in the clay, sandy, and calcareous soils, respectively) (Fig. 5). Also, although *Alphaproteobacteria* comprised the most dominant class in the metagenomic data sets, *Bacilli* made up the most frequent class found in the cultivable collection.

For LB medium, the effect of gelling agents in recovering diverse types of phyla varied, depending on the soil type. For instance, *Actinobacteria* comprised the second-largest phylum detected in the metagenomic data sets of the three soils (15.9 to 17.5%), and a similar proportional trend was observed in the culture collection obtained on LB medium solidified with Phytagel from the sandy and calcareous soils (20 and 15.4%, respectively), whereas no *Actinobacteria* were detected on this medium using the same protocol from clay soil. Conversely, a higher proportion (33.3%) was detected in the culture collection recovered on LB medium solidified with Gelrite from the clay soil, but no *Actinobacteria* were evident following the same protocol using sandy and calcareous soils. However, for Jensen medium, regardless of the soil type, the results indicated that the cultivable fractions of bacteria obtained on agar medium using the PS protocol (SJM agar) were more similar to the data from the cultivation-independent method than the bacteria obtained using other medium preparations. Based on the comparison to the metagenomic analyses of the three soil types, we concluded that the SJM agar medium tended to be less biased in terms of bacterial isolate cultivation than the other culture preparations/protocols.

Finally, we obtained cultivable isolates that were detected only using the plate-culturing techniques, and were not found in the metagenomic data sets. These isolates were identified as *Kocuria* and *Pseudarthrobacter* spp. (in the clay soil), *Microbacterium* and *Psychrobacter* spp. (in the sandy soil), and *Aureimonas* and *Variovorax* spp. (in the calcareous soil).

### PGP properties of cultivable strains.

**FIG 6:**
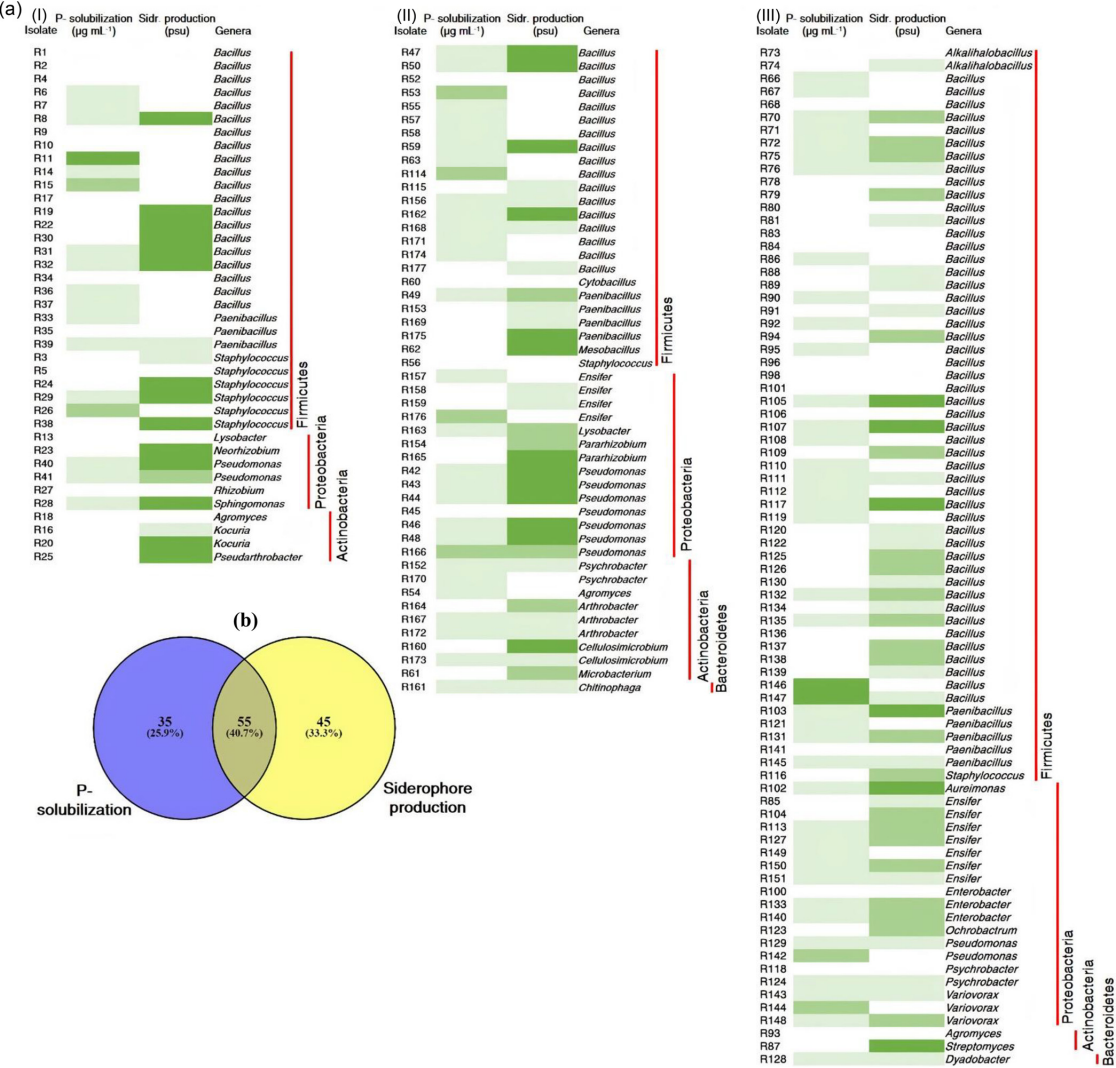
PGP characteristics of cultivable isolates. (a) Heat map of PGP traits of bacterial strains isolated from the three soil types (I) clay soil from Luxor, (II) sandy soil from Minya, and (III) calcareous soil from Nubaria. The white cells: no production; light green cells: low production; green cells: medium production; dark green cells: high production. (b) Venn-Diagram showing the isolates that presented each of the possible combinations for the two PGP evaluated traits. Values are presented as the absolute number of isolates and also as percentages (in parentheses).

Because most of the identified microbial genera in the wheat microbiomes from the three different sites are known to exhibit PGP traits—for example, *Bacillus*, *Paenibacillus*, and Pseudomonas—we screened the culture collection for two traits that are commonly associated with PGP activities. We tested for phosphate solubilization and siderophore production because each one is associated with plant nutrition and also linked to biofertilization, phytostimulation, and biocontrol. These PGP traits were evaluated *in vitro* on 166 cultivable strains that were isolated from the rhizosphere of wheat plants at the three sites (Fig. 6). The results showed that 60% were siderophore positive in plate assays, and 54% exhibited phosphate solubilization ability based on the use of agar-solidified, conventional Pikovskaya medium (31). Out of the 166 bacterial strains recovered from the rhizosphere so far, 55 exhibited both phenotypes and were consistently found in the three environments (16, 35, and 49% in the clay, sandy, and calcareous soil types, respectively). Of those, 38% were identified as *Bacillus*, 16% as Pseudomonas, and 11% as *Paenibacillus*. The rest of these isolates were classified as *Arthrobacter*, *Aureimonas*, *Cellulosimicrobium*, *Chitinophaga*, *Dyadobacter*, *Ensifer*, Enterobacter, *Sphingomonas*, Staphylococcus, and *Variovorax*. Interestingly, 81% of the analyzed isolates exhibited at least one of the two evaluated PGP traits.

## DISCUSSION

Many factors influence the culturability of microbes, such as the culture medium (e.g., medium composition and solidifying agents), growth conditions, and other cultivation techniques (21, 32, 33). Any modifications of these approaches may promote the growth of taxa that did not grow under the original conditions (34). For this purpose, multiple strategies were implemented in this study to increase the culturability percentages of bacterial communities associated with the rhizosphere of wheat plants from soils with different characteristics. Generally, we found that the gelling agent and slight changes in medium preparation were the key drivers for structuring the cultured communities in terms of viable cell counts and diversity (Fig. 1 and 2). Replacing agar with Phytagel in the case of LB medium increased CFU counts ranging from 30 to 180% in three soil samples. Similarly for the Jensen medium experiments, where agar was replaced with Phytagel and the PS protocol was used (SJM Phytagel), 61 to 273% and 10 to 127% more CFU counts compared to the JM agar medium (PT protocol) and SJM agar medium (PS protocol), respectively, were measured. Our findings are in agreement with previous publications supporting the use of gellan gum as a solidifying agent because it resulted in higher CFU counts and greater culturability of bacteria isolated from soil (32, 35) or seawater (18) than agar. The variation in culturability observed between gellan gum substrates and agar-based substrates may be due to the possibility that the two solidifying agents are composed of different sugars, each supporting the growth of specific phylotypes of microorganisms (18, 27). However, other reasons, including the accumulation of inhibitory substances or changes in components during autoclaving, may also be responsible for decreased growth in agar. Also, too high of an agar concentration is reported to limit microbial growth (36).

Concerning the method of medium preparation, the results showed that Jensen substrates when prepared using the PS protocol amended with the three different solidifying agents gave a higher colony count than the Jensen agar medium using the PT protocol (Fig. 1). Furthermore, the bacterial diversity obtained using PS medium was more reflective of the original community in the three soil samples than that obtained with PT medium (Fig. 2), again demonstrating that the PS protocol is more effective than the PT for isolating diverse genera of bacteria. This could be due to the inhibitory effect of H_2_O_2_ generated from the autoclaving of the gelling agents and phosphate together (20), and indeed separate autoclaving of the gelling agents and phosphate buffer resulted in much lower H_2_O_2_ concentrations and increased culturability of microorganisms (20, 28). Our data are also in agreement with previous publications, which reported that the PS technique is very effective for cultivating recalcitrant and novel bacterial taxa from various environments (8, 17, 21). Remarkably, isolates classified into *Ochrobactrum* and *Neorhizobium* were recovered only on Jensen medium solidified with agar following the PT protocol. We do not have a clear explanation of this finding, but these isolates may require H_2_O_2_ or unknown chemicals generated during autoclaving of phosphate and agar together for their cultivability.

Among the total OTUs revealed from the metagenomic data sets in the three analyzed soils, fewer than 1% of bacterial communities associated with the rhizosphere of wheat plants grew on LB medium and Jensen medium solidified with agar following standard protocols (LB agar and JM agar) (see Fig. S2 in the supplemental material). The modified medium preparations, including the use of alternative gelling agents, increased the percentage of the recovered bacterial genera from 1.86% to 2.52%, depending on the soil type (Fig. S2), supporting the conclusion that large numbers of bacteria remain uncultured (11).

We also observed a clear bias in results that can occur due to modifications in the method of medium preparation or addition of solidifying agents. For example, using the traditional amended agar medium (LB agar or JM agar), only one actinobacterial strain assigned to *Kocuria* sp. was recovered. However, the improved culture methods identified a complex actinobacterial community, including 6 actinobacterial genera (*Agromyces*, *Arthrobacter*, *Cellulosimicrobium*, *Microbacterium*, *Pseudarthrobacter*, and *Streptomyces* spp.) in addition to *Kocuria* spp. Similar to our findings, Adam et al. (22) obtained difficult-to-culture *Actinobacteria*—classified into *Agromyces*, *Amycolatopsis*, *Kocuria*, *Micrococcus*, *Micromonospora*, *Nocardia*, *Rhodococcus*, and *Streptomycetes* spp.—on ISP medium using the PS protocol, whereas only *Streptomyces* spp. were detected on the PT medium.

Although the cultivation-independent approach provides deep knowledge of viable bacterial communities from various environmental sources, it has many limitations. For example, some of the cultivable bacteria cannot be detected based on primer mismatches, or the diversity of certain taxa may be overestimated (37). In the present study, some of the isolated bacteria could not be detected in the metagenomic analyses, perhaps due to the bias of the selected primers or sequencing depth (34). Our data are in agreement with those of other researchers who found that certain genera of cultivable bacteria from soil (38) or marine sediments (34) were not detected using the amplicon sequencing method.

The metagenomic analyses characterizing the wheat rhizosphere revealed that *Proteobacteria*, *Actinobacteria*, *Chloroflexi*, *Bacteroidetes*, and *Firmicutes* were the dominant phyla residing in this rhizosphere in the three different soil samples. A similar phylum profile was previously described in the wheat rhizosphere microbiome grown in different regions of the world (37, 39–41). Abundant classes observed in this study were *Alphaproteobacteria*, Deltaproteobacteria, *Actinobacteria*, *Betaproteobacteria*, *Gammaproteobacteria*, and *Bacilli*, which is consistent with other studies that described the rhizosphere communities of different wheat varieties cultivated in Poland (37) and Pakistan (40). These results supported earlier conclusions that wheat plants recruit a core microbiome that is consistent across locations with different soil and environmental characteristics (2). In an extensive study characterizing wheat rhizosphere microbiomes across eight African and European soils, Simonin et al. (3) observed that 177 taxa were consistently detected in the wheat rhizosphere, constituting a core microbiome. Interestingly, we observed OTU variation in the calcareous soil collected from the Nubaria region compared to the other soil locations. This could be explained by differences in soil parameters, especially values of pH and electrical conductivity. In support of the effect of environmental factors, the rhizospheric soil in Nubaria is more alkaline (pH 8.51) and saline (EC, 0.84 dS m^−1^) than the other soil samples. This result is consistent with previous studies demonstrating that soil pH (3, 37, 40, 42) and salinity (43) play a significant role in shaping the wheat rhizosphere microbiome.

Using different plate-culturing techniques, we obtained 166 cultivable isolates, which were highly diverse at the genus level, from the three analyzed soils of the wheat rhizosphere microbiome (Fig. 3 and Table 1). We found that a high proportion of these isolates had the potential for PGP activities for nutrient acquisition, such as siderophore production and P solubilization (Fig. 6). Most of these isolates were classified as *Bacillus*, *Paenibacillus*, Pseudomonas, Enterobacter, and *Sphingomonas* spp., all of which are well-known PGPR with beneficial effects on wheat growth and health under controlled and field conditions (44–46). However, these isolates were tested only on the original Pikovskaya medium (31), which may not be an appropriate diagnostic indicator of phosphate solubilization activity (47). Additional tests to verify phosphate solubilization ability need to be performed.

In a previous study, Sheirdil et al. (45) reported the PGPR potential of *Bacillus* and Pseudomonas strains isolated from the wheat rhizosphere based on ACC deaminase activity, P solubilization ability, and siderophore production, with the potential for improving the growth of wheat plants under low inputs of chemical fertilizers in field experiments. Our results demonstrated that isolates belonging to *Ensifer* and *Dyadobacter* spp. were also identified as promising PGPRs. Consistent with our findings, there are numerous reports describing the PGP activities of *Ensifer* sp., many of which are N_2_ fixers (48, 49); however, there is currently little evidence for *Dyadobacter* spp. acting as PGPR (50).

### Conclusion.

This study presents a detailed characterization of the wheat rhizosphere microbiome under contrasting soil conditions using improved culture-dependent and culture-independent protocols. We demonstrated that modification of the medium preparation (separate sterilization of phosphate and gelling agents during medium preparation) and the use of alternative solidifying agents effectively improved the culturability of bacterial communities associated with the wheat rhizosphere. We believe that different culture strategies should be applied in parallel to bring more bacteria into culture and to discover new PGPR candidates, which are the key players for future sustainable agriculture. We determined that numerous isolates from our culture collection exhibited multiple PGP traits. Therefore, future studies are needed to evaluate their efficiency in promoting plant growth under controlled and field conditions. The diversity of bacterial communities recovered using various culture protocols was substantially lower than that obtained with the cultivation-independent method; thus, a combination of two approaches is required to provide a deeper knowledge of the soil microbiome and shed light on both cultured and uncultured bacteria that are correlated with plant health and productivity.

## MATERIALS AND METHODS

### Study area and soil sampling.

Rhizosphere soil of wheat plants (variety Misr 1) was collected in triplicate from three regions: the Nubaria region (El Beheira Governorate, Lower Egypt), Mallawi (Minya Governorate, Middle Egypt), and El Matanah (Luxor Governorate, Upper Egypt) (see Fig. S3 in the supplemental material). The soil samples were collected when the wheat plants reached the tillering stage (50 to 60 days after sowing). The soil was gathered from the wheat rhizosphere by gently removing the plants and obtaining the soil attached to the roots, followed by a thorough mixing that yielded a composite sample from each site. A total of 0.5 kg of the soil from each of the three sites was sent to UCLA for metagenomic analysis. Physical and chemical analyses of the collected soil samples were made (Table S1) (51).

### Analysis of bacterial diversity using culture-dependent techniques.

For each composite soil sample, 10 g of soil was suspended in 90 ml of sterile distilled water and vortexed thoroughly for 1 h at 150 rpm. From this stock solution, serial dilutions were performed to 10^−7^. Three replicates of 100 μl from dilutions of 10^−3^ to 10^−7^ were plated onto either nonselective Luria-Bertani (LB) medium (52) (48501; Serva, Heidelberg, Germany) or selective Jensen medium (53), a culture medium that supports the growth of some nitrogen-fixing bacteria (20.0 g liter^−1^ sucrose, 1.0 g liter^−1^ K_2_HPO_4_, 0.5 g liter^−1^ MgSO_4_ · 7H_2_O, 0.5 g liter^−1^ NaCl, 0.1 g liter^−1^ FeSO_4_ · 7H_2_O, 2.0 g liter^−1^ CaCO_3_, 0.005 g liter^−1^ Na_2_MoO_4_H_2_O). The two media were solidified with either 1.5% agar (11392; Serva, Heidelberg, Germany), 1% Phytagel (P8169; Sigma-Aldrich, USA), or 0.75% Gelrite (22168; Serva, Heidelberg, Germany). For LB medium, individual solidifying agents were autoclaved together with the medium components. In contrast, as described earlier, Jensen medium (JM) was prepared by using two different protocols, PS and PT, to minimize hydrogen peroxide formation. The results were then compared with traditional Jensen medium solidified by agar with all of the components autoclaved together (PT). After inoculation, the petri dishes were incubated at 28°C for 6 days. The number of CFU on each plate was counted during the incubation. Only plates with 20 to 200 CFU were recorded. Bacterial colonies with different shapes, sizes, and colors were purified separately by subculture on their respective media.

Total genomic DNA from individual colonies was isolated using a GeneJet Genomic DNA purification kit (Fermentas, Thermo Scientific, EU). The procedures were done according to the manufacturer’s instructions. Bacterial 16S rRNA genes were amplified using the 27F/1492R primers (54, 55). PCR was performed using the standard reaction mixture (25 µl) containing 1× PCR buffer, 1.5 mM MgCl_2_, 5% dimethyl sulfoxide, 200 mM each deoxynucleoside triphosphates (dNTPs), 15 pmol of each primer, 1 U of *Taq* polymerase enzyme (Promega Corporation, Madison, WI, USA), and 50 ng of DNA template. Thermal cycling conditions were as follows: initial denaturation at 94°C for 5 min, followed by 30 cycles of 94°C for 1 min, 55°C for 1 min, and 72°C for 1 min, and a final elongation at 72°C for 10 min. 16S rRNA gene sequencing was performed at Macrogen, Inc., South Korea. Sequence reads were edited and assembled using DNASTAR software (Lasergene, Madison, WI, USA). The taxonomical identification of bacterial isolates was done to the genus level by BLAST analysis of partial 16S rRNA gene sequences at the GenBank (http://www.ncbi.nlm.nih.gov), EzBioCloud (http://eztaxon-e.ezbiocloud.net), and Ribosomal Database Project (RDP) (https://rdp.cme.msu.edu) databases. The sequences were aligned using ClustalW version 1.8 (56) and were subjected to phylogenetic analyses. Phylogenetic trees were reconstructed using the maximum likelihood (ML) algorithm (57) in MEGA X version 10 (58) using the Jukes-Cantor model. Bootstrap support (BT) for each node was evaluated with 1,000 replicates.

### Characterization of the cultured bacteria for PGP traits. (i) Phosphate (P) solubilization.

A quantitative analysis of P solubilization activity was performed using the molybdate blue color method (59). Briefly, bacterial isolates were inoculated in 25 ml Pikovskaya broth medium (31) and incubated for 7 days at 28°C with shaking at 150 rpm. Bacterial cultures were centrifuged at 15,000 rpm for 30 min. The supernatant (1 ml) was mixed with 10 ml of chloromolybidic acid and the volume was made up to 45 ml with distilled water. Cholorostannous acid (0.25 ml) was added, and the volume was brought up to 50 ml with distilled water. The absorbance of the developing blue color was measured by spectrophotometry (Thermo, USA) at 600 nm. The amount of solubilized phosphate was detected using a standard curve produced with dilutions of a KH_2_PO_4_ solution (Sigma-Aldrich, USA).

### (ii) Siderophore production.

Bacterial isolates were assayed for siderophore production using a modified microplate method (60). All bacterial isolates were inoculated in LB broth medium, then 0.1 ml of overnight culture supernatant was mixed with 0.1 ml of chrome azurol S (CAS) reagent. Absorbance was measured at 630 nm against a reference consisting of 0.1 ml of uninoculated broth and 0.1 ml of CAS reagent. Siderophore production was expressed in percent siderophore units (PSU), which was calculated according to the formula siderophore production (PSU) = (A r − A s)/A r × 100, where *A_r_* is the absorbance of the reference (CAS solution and uninoculated broth) and *A_s_* is the absorbance of the sample (CAS solution and cell-free supernatant of the sample) (61).

### Analysis of bacterial diversity using culture-independent techniques.

Environmental DNA (eDNA) was isolated from 0.5-g rhizosphere soil samples collected from the Nubaria, Minya, and Luxor sites using a DNeasy PowerSoil Pro DNA isolation kit (Qiagen, Germany) following the manufacturer’s instructions. 16S rRNA amplicon library preparation and sequencing were performed by MR DNA analysis service (https://www.mrdnalab.com; MR DNA Shallowater, TX, USA). The hypervariable region V4 of the 16S rRNA gene was amplified using the universal primer pair 515F/806R with the HotStarTaq Plus master mix kit (Qiagen, Valencia, CA) under the following conditions: 94°C (3 min), followed by 30 cycles of 94°C for 30 s, 53°C for 40 s, and 72°C for 1 min, after which a final elongation step at 72°C (5 min) was performed. PCR products were purified using Ampure XP beads (Beckman Coulter, Inc., Brea, CA) and then were used to prepare an Illumina DNA library. The final amplicon libraries were sequenced on the Illumina MiSeq platform (Illumina Inc., San Diego, CA) following the manufacturer’s guidelines.

Sequence data were processed using a proprietary analysis pipeline (https://www.mrdnalab.com; MR DNA, Shallowater, TX, USA) and were clustered using the Usearch program (version 11.0.667) (https://www.drive5.com/usearch). Paired-end reads were merged, depleted of barcodes, and low-quality reads and short reads (<150 bp) were discarded. Chimeric sequences were removed and OTUs were generated. OTUs were defined by clustering at 3% divergence (97% similarity). OTUs were submitted to the RDP classifier (http://rdp.cme.msu.edu) and NCBI (www.ncbi.nlm.nih.gov) to obtain the taxonomy assignment. Finally, the generated OTUs were compiled into each taxonomic level into both “counts” and “percentage” files, where the “count” files contain the number of sequences, and the “percentage” files contain the relative percentage or proportion of sequences within each sample.

### Data availability.

The sequence data from BLAST analysis of partial 16S rRNA gene sequences from GenBank, EzBioCloud, and the RDP were submitted to the NCBI database under accession no. LC482422 to LC482500, LC481905 to LC481951, and LC481645 to LC481683 for cultivable bacteria in the wheat rhizosphere microbiome collected from the Nubaria, Minya, and Luxor sites, respectively. The metagenomic amplicon libraries were sequenced on the Illumina MiSeq platform (Illumina, Inc., San Diego, CA) following the manufacturer’s guidelines. The sequencing data from sequencing of the metagenomic amplicon libraries on the Illumina MiSeq platform were deposited in the NCBI database (https://www.ncbi.nlm.nih.gov) under accession no. MZ974967 to MZ976766 (Luxor), MZ973013 to MZ974947 (Minya), and MZ971364 to MZ972785 (Nubaria).
