# Supplementary material for: Comparative Analysis of the Cultured and Total Bacterial Community in the Wheat Rhizosphere Microbiome Using Culture-Dependent and Culture-Independent Approaches
Source: Microbiol Spectr. 2021 Oct 20;9(2):e00678-21. doi: 10.1128/Spectrum.00678-21 (PMC8528112; doi:10.1128/Spectrum.00678-21)
Supplement: SUPPLEMENTAL FILE 1 — Supplemental material. Download SPECTRUM00678-21_Supp_1_seq13.pdf, PDF file, 3.3 MB [file spectrum00678-21_supp_1_seq13.pdf]

Supplementary Material

Comparative analysis of the cultured and total bacterial community in the wheat rhizosphere microbiome using culture-dependent and culture-independent approaches

Sameh H. Youseif,[a] Fayrouz H. Abd El-Megeed,[a] Ethan A. Humm,[b] Maskit Maymon,[b] Akram H. Mohamed,[a] Saleh A. Saleh,[c] Ann M. Hirsch[b,d]

[a] Department of Microbial Genetic Resources, National Gene Bank (NGB), Agricultural Research Center (ARC), Giza 12619, Egypt

[b] Department of Molecular, Cell & Developmental Biology, University of California-Los Angeles (UCLA), 621 Charles Young Drive South, Los Angeles, CA 90095-1606, U.S.A.

[c] Agricultural Microbiology Research Department, Soils, Water and Environment Research Institute, Agricultural Research Center (ARC), Giza, Egypt

[d] Molecular Biology Institute, UCLA, 621 Charles Young Drive South, Los Angeles, CA 90095-1606, U.S.A.

TABLE S1 Physical and chemical analysis of the three collected soil samples

Property	value		
	**El Matanah, Luxor**	**Mallawi, Minya**	**Nubaria region, El Beheira**
Latitude	25°25'27.97"N	27°43'17.03"N	30°54'16.25"N
Longitude	32°32'18.17"E	30°43'20.91"E	29°52'43.59"E
Particle size distribution (%)			
Sand	21.90	88.70	54.30
Silt	32.60	5.90	25.10
Clay	45.50	5.40	20.60
Saturation percent (%)	44.50	18.20	26.20
$CaCO_3$ (%)	1.94	2.80	18.80
pH (soil paste)	8.00	7.93	8.51
E.C (dS m^{-1}, at 25ºC)	0.62	0.33	0.84
Soluble cations (meq L^{-1})			
Ca^{++}	3.10	0.90	2.59
Mg^{++}	0.80	0.64	1.55
Na^{+}	1.21	1.31	4.21
K^{+}	1.04	0.59	0.14
Soluble anions (meq L^{-1})			
$CO^{=}_3$	0.00	0.00	0.00
HCO^{-}_3	1.96	1.43	2.41
Cl^{-}	1.77	0.58	3.98
$SO^{=}_4$	2.42	1.43	2.10
Total-N (%)	0.067	0.020	0.015
Total soluble-N (mg kg^{-1})	52.20	14.30	16.40
Available-P (mg kg^{-1})	9.90	4.40	3.40
Available-K (mg kg^{-1})	331.80	180.00	127.00
Organic matter (%)	0.72	0.16	0.21
DTPA-extractable (mg kg^{-1})			
Fe	4.82	3.16	0.61
Mn	2.41	1.16	0.46
Zn	1.82	0.21	0.38
Cu	0.25	0.02	0.09

DTPA: Di-ethylene tri-amine penta acetic acid.

TABLE S2 List of isolated bacteria and their respective soil type and media preparations

No.	Isolate	Soil type	Media preparation**	No.	Isolate	Soil type	Media preparation
**1**	NGB-R1	Clay	LB-Phytagel™	**90**	NGB-R90	Calcareous	LB-Phytagel™
**2**	NGB-R2	Clay	LB-Phytagel™	**91**	NGB-R91	Calcareous	LB-Phytagel™
**3**	NGB-R3	Clay	LB-Phytagel™	**92**	NGB-R92	Calcareous	LB-Phytagel™
**4**	NGB-R4	Clay	LB-Phytagel™	**93**	NGB-R93	Calcareous	LB-Phytagel™
**5**	NGB-R5	Clay	LB-Phytagel™	**94**	NGB-R94	Calcareous	LB-Phytagel™
**6**	NGB-R6	Clay	LB-Phytagel™	**95**	NGB-R95	Calcareous	LB-Phytagel™
**7**	NGB-R7	Clay	LB-Phytagel™	**96**	NGB-R96	Calcareous	LB-Phytagel™
**8**	NGB-R8	Clay	LB-Phytagel™	**97**	NGB-R97*	Calcareous	LB-Gelrite®
**9**	NGB-R9	Clay	LB-Phytagel™	**98**	NGB-R98	Calcareous	LB-Gelrite®
**10**	NGB-R10	Clay	LB-Phytagel™	**99**	NGB-R99*	Calcareous	LB-Gelrite®
**11**	NGB-R11	Clay	LB-Phytagel™	**100**	NGB-R100	Calcareous	LB-Gelrite®
**12**	NGB-R12*	Clay	LB-Agar	**101**	NGB-R101	Calcareous	LB-Gelrite®
**13**	NGB-R13	Clay	LB-Agar	**102**	NGB-R102	Calcareous	LB-Gelrite®
**14**	NGB-R14	Clay	LB-Agar	**103**	NGB-R103	Calcareous	LB-Gelrite®
**15**	NGB-R15	Clay	LB-Agar	**104**	NGB-R104	Calcareous	LB-Gelrite®
**16**	NGB-R16	Clay	LB-Agar	**105**	NGB-R105	Calcareous	LB-Gelrite®
**17**	NGB-R17	Clay	LB-Agar	**106**	NGB-R106	Calcareous	LB-Gelrite®
**18**	NGB-R18	Clay	LB-Gelrite®	**107**	NGB-R107	Calcareous	LB-Gelrite®
**19**	NGB-R19	Clay	LB-Gelrite®	**108**	NGB-R108	Calcareous	LB-Gelrite®
**20**	NGB-R20	Clay	LB-Gelrite®	**109**	NGB-R109	Calcareous	LB-Gelrite®
**21**	NGB-R21*	Clay	JM-Agar	**110**	NGB-R110	Calcareous	LB-Gelrite®
**22**	NGB-R22	Clay	JM-Agar	**111**	NGB-R111	Calcareous	LB-Gelrite®
**23**	NGB-R23	Clay	JM-Agar	**112**	NGB-R112	Calcareous	LB-Gelrite®
**24**	NGB-R24	Clay	JM-Agar	**113**	NGB-R113	Calcareous	LB-Gelrite®
**25**	NGB-R25	Clay	JM-Agar	**114**	NGB-R114	Sandy	LB-Agar
**26**	NGB-R26	Clay	JM-Agar	**115**	NGB-R115	Sandy	LB-Agar
**27**	NGB-R27	Clay	JM-Agar	**116**	NGB-R116	Calcareous	JM-Agar
**28**	NGB-R28	Clay	JM-Agar	**117**	NGB-R117	Calcareous	JM-Agar
**29**	NGB-R29	Clay	SJM-Phytagel™	**118**	NGB-R118	Calcareous	JM-Agar
**30**	NGB-R30	Clay	SJM-Phytagel™	**119**	NGB-R119	Calcareous	JM-Agar
**31**	NGB-R31	Clay	SJM-Phytagel™	**120**	NGB-R120	Calcareous	JM-Agar
**32**	NGB-R32	Clay	SJM-Phytagel™	**121**	NGB-R121	Calcareous	JM-Agar
**33**	NGB-R33	Clay	SJM-Phytagel™	**122**	NGB-R122	Calcareous	JM-Agar
**34**	NGB-R34	Clay	SJM-Phytagel™	**123**	NGB-R123	Calcareous	JM-Agar
**35**	NGB-R35	Clay	SJM-Phytagel™	**124**	NGB-R124	Calcareous	SJM-Agar
**36**	NGB-R36	Clay	SJM-Gelrite®	**125**	NGB-R125	Calcareous	SJM-Agar
**37**	NGB-R37	Clay	SJM-Gelrite®	**126**	NGB-R126	Calcareous	SJM-Agar
**38**	NGB-R38	Clay	SJM-Gelrite®	**127**	NGB-R127	Calcareous	SJM-Agar
**39**	NGB-R39	Clay	SJM-Gelrite®	**128**	NGB-R128	Calcareous	SJM-Agar
**40**	NGB-R40	Clay	SJM-Gelrite®	**129**	NGB-R129	Calcareous	SJM-Agar
**41**	NGB-R41	Clay	SJM-Gelrite®	**130**	NGB-R130	Calcareous	SJM-Agar
**42**	NGB-R42	Sandy	LB-Agar	**131**	NGB-R131	Calcareous	SJM-Agar
**43**	NGB-R43	Sandy	LB-Agar	**132**	NGB-R132	Calcareous	SJM-Phytagel™
**44**	NGB-R44	Sandy	LB-Agar	**133**	NGB-R133	Calcareous	SJM-Phytagel™
**45**	NGB-R45	Sandy	LB-Agar	**134**	NGB-R134	Calcareous	SJM-Phytagel™
**46**	NGB-R46	Sandy	LB-Agar	**135**	NGB-R135	Calcareous	SJM-Phytagel™
**47**	NGB-R47	Sandy	LB-Agar	**136**	NGB-R136	Calcareous	SJM-Phytagel™
**48**	NGB-R48	Sandy	LB-Agar	**137**	NGB-R137	Calcareous	SJM-Phytagel™
**49**	NGB-R49	Sandy	LB-Agar	**138**	NGB-R138	Calcareous	SJM-Phytagel™
**50**	NGB-R50	Sandy	LB-Agar	**139**	NGB-R139	Calcareous	SJM-Phytagel™
**51**	NGB-R51*	Sandy	LB-Agar	**140**	NGB-R140	Calcareous	SJM-Phytagel™

**52**	NGB-R52	Sandy	LB-Agar	**141**	NGB-R141	Calcareous	SJM-Phytagel™
**53**	NGB-R53	Sandy	LB-Phytagel™	**142**	NGB-R142	Calcareous	SJM-Phytagel™
**54**	NGB-R54	Sandy	LB-Phytagel™	**143**	NGB-R143	Calcareous	SJM-Phytagel™
**55**	NGB-R55	Sandy	LB-Phytagel™	**144**	NGB-R144	Calcareous	SJM-Phytagel™
**56**	NGB-R56	Sandy	LB-Phytagel™	**145**	NGB-R145	Calcareous	SJM-Gelrite®
**57**	NGB-R57	Sandy	LB-Phytagel™	**146**	NGB-R146	Calcareous	SJM-Gelrite®
**58**	NGB-R58	Sandy	LB-Phytagel™	**147**	NGB-R147	Calcareous	SJM-Gelrite®
**59**	NGB-R59	Sandy	LB-Phytagel™	**148**	NGB-R148	Calcareous	SJM-Gelrite®
**60**	NGB-R60	Sandy	LB-Phytagel™	**149**	NGB-R149	Calcareous	SJM-Gelrite®
**61**	NGB-R61	Sandy	LB-Phytagel™	**150**	NGB-R150	Calcareous	SJM-Gelrite®
**62**	NGB-R62	Sandy	LB-Phytagel™	**151**	NGB-R151	Calcareous	SJM-Gelrite®
**63**	NGB-R63	Sandy	LB-Gelrite®	**152**	NGB-R152	Sandy	JM-Agar
**64**	NGB-R64*	Sandy	LB-Gelrite®	**153**	NGB-R153	Sandy	JM-Agar
**65**	NGB-R65*	Sandy	LB-Gelrite®	**154**	NGB-R154	Sandy	JM-Agar
**66**	NGB-R66	Calcareous	LB-Agar	**155**	NGB-R155*	Sandy	SJM-Agar
**67**	NGB-R67	Calcareous	LB-Agar	**156**	NGB-R156	Sandy	SJM-Agar
**68**	NGB-R68	Calcareous	LB-Agar	**157**	NGB-R157	Sandy	SJM-Agar
**69**	NGB-R69*	Calcareous	LB-Agar	**158**	NGB-R158	Sandy	SJM-Agar
**70**	NGB-R70	Calcareous	LB-Agar	**159**	NGB-R159	Sandy	SJM-Agar
**71**	NGB-R71	Calcareous	LB-Agar	**160**	NGB-R160	Sandy	SJM-Agar
**72**	NGB-R72	Calcareous	LB-Agar	**161**	NGB-R161	Sandy	SJM-Agar
**73**	NGB-R73	Calcareous	LB-Agar	**162**	NGB-R162	Sandy	SJM-Agar
**74**	NGB-R74	Calcareous	LB-Agar	**163**	NGB-R163	Sandy	SJM-Agar
**75**	NGB-R75	Calcareous	LB-Agar	**164**	NGB-R164	Sandy	SJM-Agar
**76**	NGB-R76	Calcareous	LB-Agar	**165**	NGB-R165	Sandy	SJM-Agar
**77**	NGB-R77*	Calcareous	LB-Agar	**166**	NGB-R166	Sandy	SJM-Agar
**78**	NGB-R78	Calcareous	LB-Agar	**167**	NGB-R167	Sandy	SJM-Phytagel™
**79**	NGB-R79	Calcareous	LB-Agar	**168**	NGB-R168	Sandy	SJM-Phytagel™
**80**	NGB-R80	Calcareous	LB-Agar	**169**	NGB-R169	Sandy	SJM-Phytagel™
**81**	NGB-R81	Calcareous	LB-Agar	**170**	NGB-R170	Sandy	SJM-Phytagel™
**82**	NGB-R82*	Calcareous	LB-Agar	**171**	NGB-R171	Sandy	SJM-Phytagel™
**83**	NGB-R83	Calcareous	LB-Agar	**172**	NGB-R172	Sandy	SJM-Gelrite®
**84**	NGB-R84	Calcareous	LB-Phytagel™	**173**	NGB-R173	Sandy	SJM-Gelrite®
**85**	NGB-R85	Calcareous	LB-Phytagel™	**174**	NGB-R174	Sandy	SJM-Gelrite®
**86**	NGB-R86	Calcareous	LB-Phytagel™	**175**	NGB-R175	Sandy	SJM-Gelrite®
**87**	NGB-R87	Calcareous	LB-Phytagel™	**176**	NGB-R176	Sandy	SJM-Gelrite®
**88**	NGB-R88	Calcareous	LB-Phytagel™	**177**	NGB-R177	Sandy	SJM-Gelrite®
**89**	NGB-R89	Calcareous	LB-Phytagel™				

* Bacterial colonies failed to grow.

**Agar, Phytagel™, and Gelrite® were used in the following concentration 1.5%, 1%, and 0.75% respectively.

JM: Jensen medium prepared by autoclaving phosphates and solidifying agent together. SJM: Jensen medium prepared by autoclaving phosphates and solidifying agent separately. Clay, sandy, and calcareous soils were collected from Luxor, Minya, and Nubaria sites, respectively.

TABLE S3 Distribution of bacterial phyla and their percentages in the three soils based on the metagenomic analyses

No.	Phylum	Luxor (Clay soil)	Minya (sandy soil)	Nubaria (calcareous soil)
**1**	Acidobacteria	4.323	3.291	3.116
**2**	Actinobacteria	17.189	17.527	15.963
**3**	Armatimonadetes	0.045	0.051	0.019
**4**	Bacteroidetes	3.862	4.099	11.684
**5**	Candidatus Bipolaricaulota	0.000	0.007	0.000
**6**	Candidatus Melainabacteria	0.002	0.002	0.000
**7**	Candidatus Saccharibacteria	0.004	0.002	0.030
**8**	Candidatus Tectomicrobia	0.054	0.073	0.560
**9**	Chlamydiae	0.002	0.000	0.048
**10**	Chlorobi	0.007	0.000	0.005
**11**	Chloroflexi	10.214	13.471	4.983
**12**	Chlorophyta	0.000	0.000	0.087
**13**	Cyanobacteria	0.509	0.411	0.841
**14**	Deinococcus-Thermus	0.073	0.246	0.152
**15**	Elusimicrobia	0.004	0.000	0.012
**16**	Fibrobacteres	0.326	0.243	0.129
**17**	Firmicutes	4.627	7.950	10.257
**18**	Fusobacteria	0.009	0.002	0.004
**19**	Gemmatimonadetes	2.641	2.772	2.471
**20**	Ignavibacteriae	0.028	0.042	0.062
**21**	Nitrospinae	0.000	0.000	0.004
**22**	Nitrospirae	0.584	0.476	0.576
**23**	Planctomycetes	3.452	4.700	4.706
**24**	Proteobacteria	40.757	36.572	32.533
**25**	Rhodothermaeota	0.000	0.009	0.014
**26**	Spirochaetes	0.000	0.000	0.012
**27**	Synergistetes	0.000	0.000	0.005
**28**	Tenericutes	0.019	0.018	0.048
**29**	Thermotogae	0.000	0.000	0.009
**30**	Verrucomicrobia	2.464	1.964	3.079

TABLE S4 Distribution of bacterial classes and their percentages in the three soils based on the metagenomic analyses

No.	Class	Luxor (clay)	Minya (sandy)	Nubaria (calcareous)	No.	Class	Luxor (clay)	Minya (sandy)	Nubaria (calcareous)
**1**	Acetothermiia	0.000	0.007	0.000	**36**	Gammaproteobacteria	4.543	7.171	8.674
**2**	Acidimicrobiia	3.762	1.128	0.984	**37**	Gemmatimonadetes	2.641	2.772	2.471
**3**	Acidithiobacillia	0.000	0.020	0.021	**38**	Gloeobacteria	0.000	0.009	0.000
**4**	Acidobacteriia	0.000	2.581	2.464	**39**	Holophagae	1.130	0.502	0.505
**5**	Actinobacteria	10.705	11.852	10.405	**40**	Ignavibacteria	0.028	0.042	0.062
**6**	Alphaproteobacteria	14.403	15.723	11.062	**41**	Ktedonobacteria	0.022	0.004	0.000
**7**	Anaerolineae	3.105	3.902	1.791	**42**	Melainabacteria	0.002	0.002	0.000
**8**	Ardenticatenia	0.007	0.000	0.000	**43**	Mollicutes	0.019	0.018	0.048
**9**	Armatimonadia	0.002	0.015	0.000	**44**	Negativicutes	0.000	0.000	0.002
**10**	Bacilli	1.865	6.193	7.010	**45**	Nitriliruptoria	0.250	0.197	0.189
**11**	Bacteriodetes	0.050	0.000	0.000	**46**	Nitrospinia	0.000	0.000	0.004
**12**	Bacteroidia	1.029	0.429	3.842	**47**	Nitrospira	0.584	0.476	0.576
**13**	Betaproteobacteria	8.609	5.439	4.040	**48**	Oligoflexia	0.041	0.230	0.254
**14**	Blastocatellia	0.140	0.208	0.147	**49**	Opitutae	1.343	0.509	1.367
**15**	Caldilineae	0.261	0.659	0.375	**50**	Oscillatoriophycideae	0.000	0.104	0.000
**16**	Candidatus Brocadiae	0.032	0.049	0.152	**51**	Phycisphaerae	0.688	0.484	0.429
**17**	Chitinophagia	0.908	1.163	1.477	**52**	Planctomycetia	2.732	4.167	4.125
**18**	Chlamydiia	0.002	0.000	0.048	**53**	Rhodothermaceae	0.000	0.000	0.048
**19**	Chlorobia	0.007	0.000	0.005	**54**	Rhodothermia	0.000	0.009	0.014
**20**	Chloroflexia	3.577	4.353	1.168	**55**	Rhodothermaeota	0.002	0.000	0.000
**21**	Chthonomonadetes	0.032	0.024	0.009	**56**	Rubrobacteria	2.527	1.203	0.954
**22**	Clostridia	2.497	1.659	2.782	**57**	Saccharibacteria	0.004	0.002	0.030
**23**	Conoidasida	0.000	0.009	0.000	**58**	Saprospiria	0.060	0.069	0.413
**24**	Cyanophyceae	0.509	0.299	0.841	**59**	Spartobacteria	0.028	0.252	0.071
**25**	Cytophagia	1.020	1.807	4.098	**60**	Sphingobacteriia	0.449	0.338	1.480
**26**	Dehalococcoidia	1.128	1.405	0.956	**61**	Spirochaetia	0.000	0.000	0.012
**27**	Deinococci	0.073	0.246	0.152	**62**	Synergistia	0.000	0.000	0.005
**28**	Deltaproteobacteria	13.014	7.967	8.437	**63**	Tectomicrobia	0.000	0.073	0.560
**29**	Elusimicrobia	0.004	0.000	0.012	**64**	Thermoanaerobacterales	0.043	0.000	0.000
**30**	Epsilonproteobacteria	0.147	0.022	0.044	**65**	Thermoleophilia	2.999	3.148	3.431
**31**	Erysipelotrichia	0.216	0.077	0.436	**66**	thermomicobia	2.113	3.148	0.694
**32**	Fibrobacteria	0.326	0.243	0.129	**67**	Thermotogae	0.000	0.000	0.009
**33**	Fimbriimonadia	0.011	0.011	0.011	**68**	Tissierellia	0.006	0.020	0.026
**34**	Flavobacteria	0.343	0.292	0.325	**69**	Ulvophyceae	0.000	0.000	0.087
**35**	Fusobacteriia	0.009	0.002	0.004	**70**	Verrucomicrobiae	1.093	1.203	1.641

TABLE S5 Variability of the top genera detected in the three soils, their percentages, and taxonomy based on the metagenomic analyses

**No.**	**Genus**	**Luxor (clay soil)**	**Minya (sandy soil)**	**Nubaria (calcareous soil)**	**Taxonomy (class; phylum)**
**1**	*Pelobacter*	6.399	3.568	2.742	Deltaproteobacteria; Proteobacteria
**2**	*Bacillus*	1.317	4.497	5.328	Bacilli; Firmicutes
**3**	*Candidatus nitrososphaera*	5.768	-	-	Nitrososphaeria; Thaumarchaeota
**4**	*Rhodopseudomonas*	3.228	0.829	0.442	Alphaproteobacteria; Proteobacteria
**5**	*Gemmatimonas*	2.641	2.772	2.471	Gemmatimonadetes; Gemmatimonadetes
**6**	*Acidobacterium*	2.391	2.031	1.934	Acidobacteriia; Acidobacteria
**7**	*Nitrosospira*	2.279	0.869	0.392	Betaproteobacteria; Proteobacteria
**8**	*Rubrobacter*	2.197	1.068	0.526	Rubrobacteria; Actinobacteria
**9**	*Chloroflexus*	1.928	2.955	0.938	Chloroflexia; Chloroflexi
**10**	*Arthrobacter*	0.821	4.371	1.185	Actinomycetia; Actinobacteria
**11**	*Methylocaldum*	0.004	2.688	0.005	Gammaproteobacteria; Proteobacteria
**12**	*Sphaerobacter*	1.330	2.564	0.622	Thermomicrobia; Chloroflexi
**13**	*Levilinea*	0.699	2.143	0.723	Anaerolineae; Chloroflexi
**14**	*Cytophaga*	0.218	0.358	3.328	Cytophagia; Bacteroidetes
**15**	*Steroidobacter*	1.046	1.141	3.187	Gammaproteobacteria; Proteobacteria
**16**	*Ohtaekwangia*	0.490	0.270	3.076	Cytophagia; Bacteroidetes
**17**	*Streptomyces*	0.352	0.352	2.014	Actinomycetia; Actinobacteria

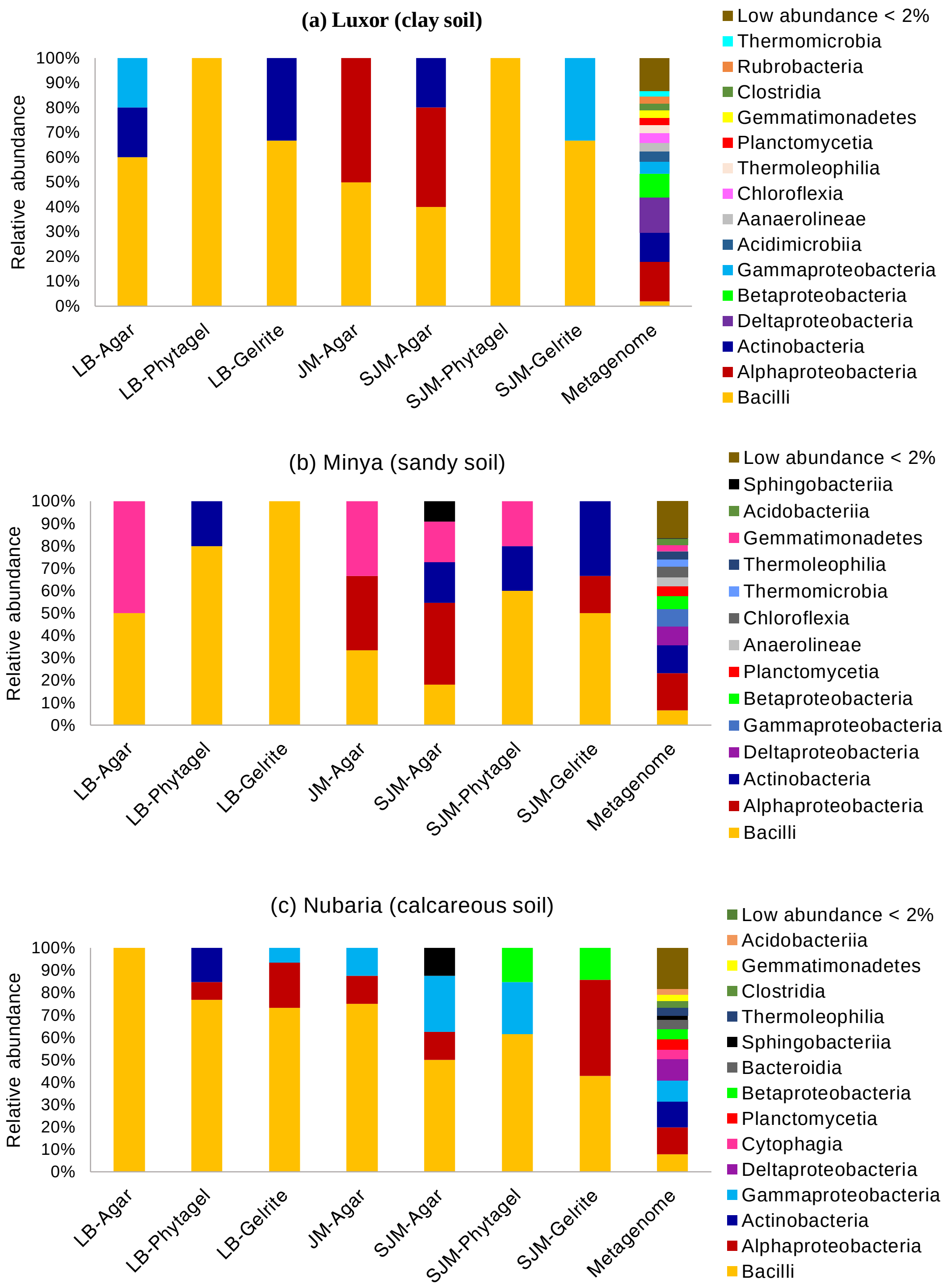

FIG S1 Comparison of metagenomic (culture-independent) and cultured bacteria datasets at the class level distributed in the three rhizospheric soil samples (a) Luxor, (b) Minya and (c) Nubaria of *T. aestivum* L. plants. JM (Jensen medium prepared by autoclaving phosphate and solidifying agent together), SJM (Jensen medium prepared by autoclaving phosphates and solidifying agent separately).

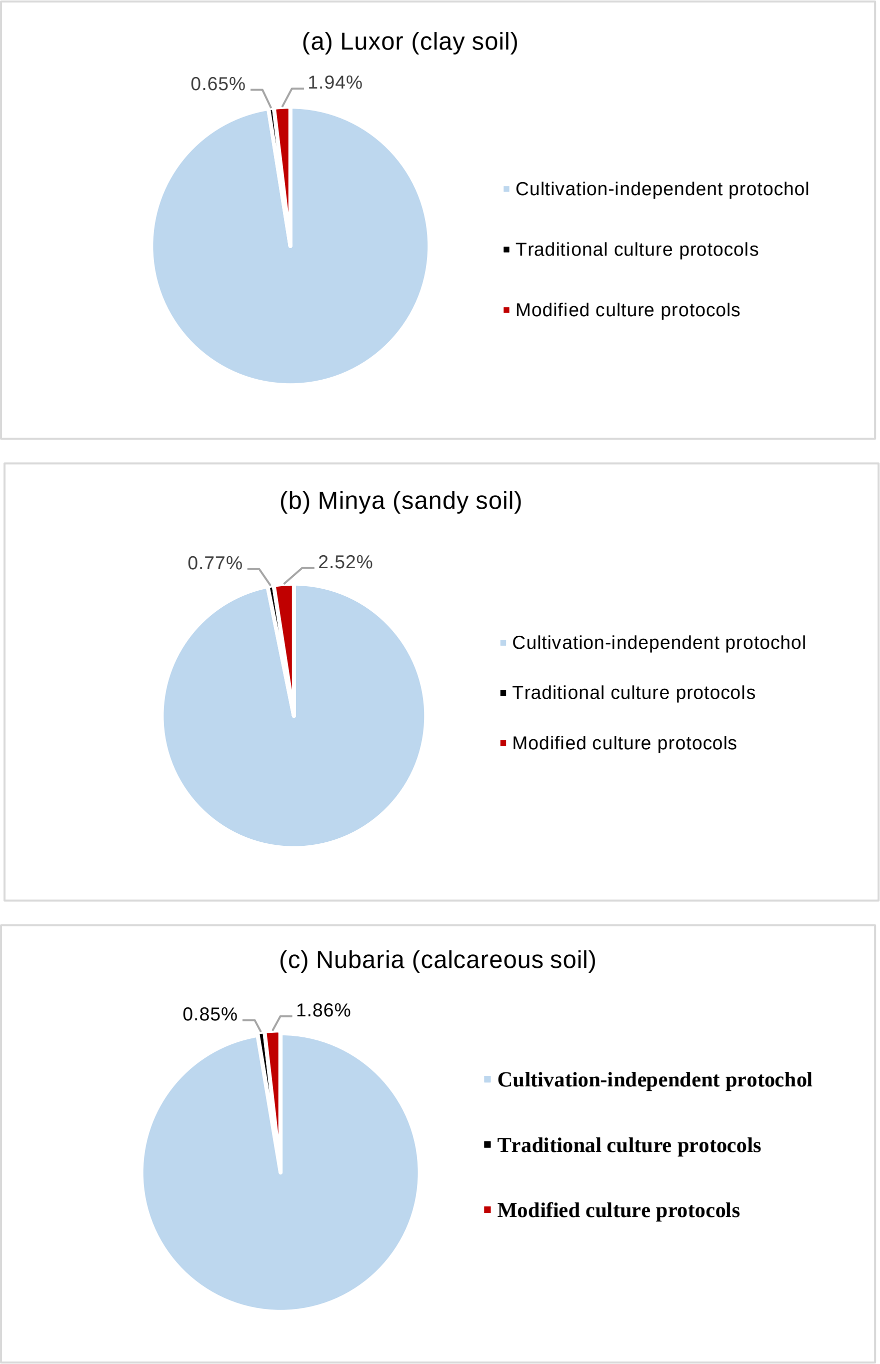

FIG S2 The percentages of cultured genera from the three analyzed soils obtained on LB and Jensen media using the standard culture protocols (LB agar and JM-agar) and the modified media preparations used in this study (LB-phytagel, LB-gelrite, SJM-agar, SJM-phytagel, and SJM-gelrite) compared to the metagenomic datasets.

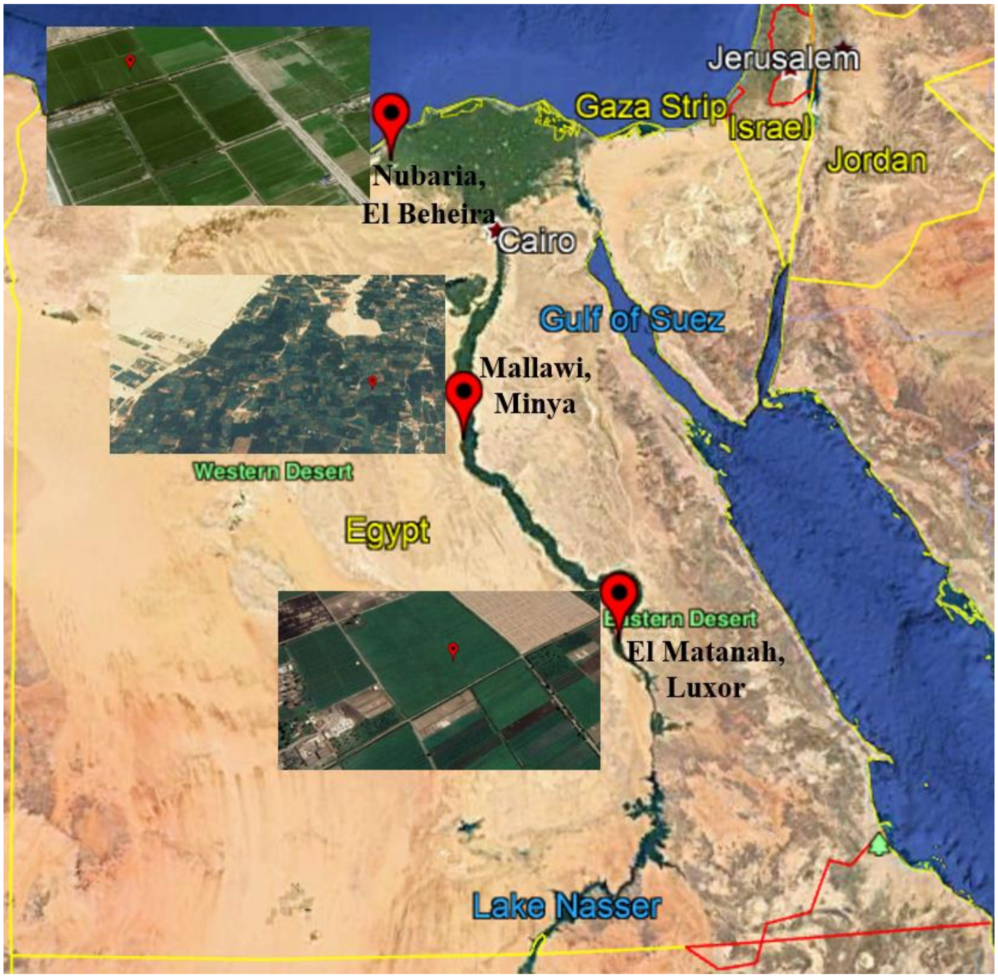

FIG S3 GIS map of the three soil sites generated by Google© Earth Pro 7.1.7.2600
